# The Cerebroplacental–Renal Ratio as a Novel Doppler-Based Marker of Adverse Perinatal Outcome in Pregnancy-Induced Hypertension

**DOI:** 10.3390/medicina62091806

**Published:** 2026-09-19

**Authors:** Yücel Kaya, Kübra Kurt Bilirer, Aybekcan Batman, Burcu Çiçek, İlteriş Yaman, Ali Selçuk Yeniocak, Can Tercan, Karolin Ohanoglu Cetinel, Damla Yasemin Yenliç Kaya, Gülseren Polat

**Affiliations:** 1 Division of Perinatology, Department of Obstetrics and Gynecology, Basaksehir Cam and Sakura City Hospital, Istanbul 34480, Turkey; kubrakbilirer@gmail.com (K.K.B.); batman.aybek.can@gmail.com (A.B.); burcu35cicek25@gmail.com (B.Ç.); 2 Division of Gynecologic Oncology Surgery, Department of Obstetrics and Gynecology, Basaksehir Cam and Sakura City Hospital, Istanbul 34480, Turkey; drilterisyaman@gmail.com; 3 Department of Obstetrics and Gynecology, Basaksehir Cam and Sakura City Hospital, Istanbul 34480, Turkey; a.s.yeniocak@hotmail.com (A.S.Y.); cntrcn89@gmail.com (C.T.); karolette@gmail.com (K.O.C.); 4 Department of Obstetrics and Gynecology, Medipol University Esenler Hospital, Istanbul 34250, Turkey; damlayenlic1@gmail.com; 5 Department of Obstetrics and Gynecology, School of Medicine, Istanbul Medipol University, Istanbul 34214, Turkey; gulserenpolat@gmail.com

**Keywords:** pre-eclampsia, ultrasonography, renal artery, pregnancy outcome, pregnancy complications

## Abstract

*Background and Objectives:* To evaluate the association between the newly defined cerebroplacental–renal ratio (CPRR) and adverse perinatal outcome (APO) in pregnancy-induced hypertension (PIH), and to compare its discriminative performance with the cerebroplacental ratio (CPR) and cerebroplacental–uterine ratio (CPUR). *Materials and Methods:* This prospective, single-center cohort study included 105 women with PIH (preeclampsia, *n* = 63; gestational hypertension, *n* = 42). Doppler assessment was performed at ≥34 + 0 weeks and within 7 days before delivery. CPR was calculated as middle cerebral artery pulsatility index (PI)/umbilical artery PI, CPUR as CPR/mean uterine artery PI, and CPRR as CPR/right renal artery PI. APO was defined by the presence of any of the following: non-reassuring fetal status, a 5 min Apgar score <7, neonatal intensive care unit admission, or neonatal death. Receiver operating characteristic analysis and multivariable logistic regression adjusted for gestational age at ultrasound assessment and diagnosis group were performed. *Results:* APO occurred in 37 pregnancies (35.2%). CPUR and CPRR were significantly lower in the APO group, whereas CPR did not differ significantly. CPRR showed a higher apparent AUC for APO than CPR and CPUR (AUC 0.786, 95% CI 0.698–0.875), with significantly higher AUCs on pairwise DeLong comparisons. After adjustment, CPRR was the only Doppler index independently associated with APO (adjusted OR 1.423 per 0.1-unit decrease; 95% CI 1.162–1.743; *p* = 0.001). *Conclusions:* CPRR was independently associated with APO in PIH and showed higher discriminative performance than CPR and CPUR; however, these findings require validation in independent, multicenter studies before clinical application.

## 1. Introduction

Pregnancy-induced hypertension (PIH), encompassing preeclampsia and gestational hypertension, is a major obstetric complication closely associated with uteroplacental dysfunction and adverse perinatal outcomes [1,2]. It affects approximately 5–10% of pregnancies and may lead to life-threatening maternal multiorgan involvement and fetal compromise [3,4]. Abnormal placentation, followed by placental ischemia and systemic endothelial dysfunction, underlies this disease spectrum [5,6]. These mechanisms concomitantly impair uteroplacental perfusion and fetal oxygenation, thereby increasing both maternal and perinatal risk.

Antenatal Doppler assessment is a principal tool for monitoring uteroplacental insufficiency and fetal hemodynamic adaptation. The cerebroplacental ratio (CPR) is an established Doppler index that reflects fetal blood flow redistribution and the brain-sparing pattern that develops in response to placental dysfunction [7]. In PIH cohorts, CPR is frequently used to predict adverse perinatal outcome (APO) [8,9]. However, this index largely confines the assessment to fetal circulatory redistribution and placental vascular resistance.

The cerebroplacental–uterine ratio (CPUR), first described by MacDonald et al., incorporates maternal uteroplacental vascular resistance into the assessment of fetoplacental hemodynamic adaptation [10]. CPUR has been reported to show greater discriminative performance than CPR in predicting APO among cases of PIH [11]. Because uterine artery Doppler measurements primarily reflect local uteroplacental perfusion, they may not fully represent the systemic maternal vascular component of the disease.

In contrast, preeclampsia is a systemic endothelial disorder whose vascular involvement extends beyond the uteroplacental unit to maternal organs, including the kidneys [12]. Renal glomerular endotheliosis disrupts capillary perfusion and reduces renal plasma flow [13]. Consistent with this pathophysiology, maternal renal artery (RA) Doppler indices—particularly peak systolic velocity and pulsatility index (PI)—have been shown to be significantly higher in pregnancies complicated by PIH than in normotensive pregnancies [14,15]. However, the available evidence remains heterogeneous. A systematic review and meta-analysis found no consistent overall difference in renal arterial RI or PI between preeclamptic and healthy pregnancies, although some venous renal Doppler parameters appeared to show more consistent alterations [16]. Despite this heterogeneity, maternal renal Doppler may still provide systemic information distinct from, and complementary to, the uteroplacental signal, thereby supporting its integration with fetoplacental indices rather than its use in isolation.

In this context, we defined the cerebroplacental–renal ratio (CPRR) as a novel Doppler index that integrates fetal cerebroplacental adaptation with maternal renal vascular resistance. Our primary objective was to investigate the association between this index and APO in pregnancies complicated by PIH. The secondary objective was to compare the discriminative performance of CPRR with that of CPR and CPUR. We hypothesized that CPRR would be associated with APO and that this association might be stronger than the associations observed for CPR and CPUR.

## 2. Materials and Methods

### 2.1. Study Design and Setting

We conducted this prospective, single-center observational cohort study at the Perinatology Clinic of Başakşehir Çam ve Sakura City Hospital, a tertiary referral center. Data collection was performed between June 2025 and May 2026. The research protocol was developed in accordance with the Declaration of Helsinki and approved by the local ethics committee (Approval No. KAEK-11/26.03.2025.112). Written informed consent was obtained from all participants before clinical data collection and ultrasonographic examinations. In addition, the methodological design and reporting adhered to the Strengthening the Reporting of Observational Studies in Epidemiology (STROBE) guidelines [17].

### 2.2. Participants

The study cohort comprised consecutive pregnant women diagnosed with PIH during the study period, including cases of preeclampsia and gestational hypertension. Gestational hypertension was defined as systolic blood pressure ≥ 140 mmHg and/or diastolic blood pressure ≥ 90 mmHg on two measurements obtained at least four hours apart after 20 weeks of gestation in a previously normotensive pregnant woman, in the absence of proteinuria or signs of maternal organ dysfunction attributable to preeclampsia. Preeclampsia was defined as the presence of proteinuria (≥300 mg/24 h, a protein/creatinine ratio ≥ 0.3, or dipstick ≥ 2+ when quantitative methods were unavailable) accompanying these hypertensive criteria, or, in the absence of proteinuria, new-onset hypertension accompanied by at least one of the following: thrombocytopenia (platelet count < 100,000/µL), renal dysfunction (serum creatinine > 1.1 mg/dL or a doubling of the baseline serum creatinine concentration in the absence of other renal disease), impaired liver function (elevation of transaminases to more than twice the upper limit of normal), pulmonary edema, a new-onset headache that was unresponsive to medication and could not be explained by alternative diagnoses, or visual symptoms [18].

Patients were included if they had a live singleton pregnancy complicated by preeclampsia or gestational hypertension, underwent Doppler assessment at ≥34 + 0 weeks of gestation, and delivered within 7 days of the Doppler assessment. Exclusion criteria comprised structural or chromosomal fetal anomalies; multiple gestation; suspected congenital infection; abnormal ductus venosus (DV) Doppler findings or umbilical artery absent/reversed end-diastolic flow detected during follow-up; a history of chronic systemic disease, including chronic hypertension, chronic kidney disease, and pregestational diabetes mellitus; other pregnancy complications, such as premature rupture of membranes, chorioamnionitis, or intrahepatic cholestasis; smoking, alcohol use, or substance use; and incomplete clinical or follow-up data.

### 2.3. Variables

APO, the primary outcome, was defined as at least one of the following: non-reassuring fetal status, a 5 min Apgar score < 7, neonatal intensive care unit (NICU) admission, or neonatal death. For the analyses, cases were categorized into “with APO” and “without APO” groups according to the presence of APO.

The main predictor variable was CPRR, which was defined in the present study. For comparative evaluation, CPR and CPUR were analyzed as secondary Doppler predictors. In addition, umbilical artery pulsatility index (UA PI), middle cerebral artery pulsatility index (MCA PI), mean uterine artery pulsatility index (UtA PI), and right renal artery pulsatility index (RA PI) were recorded as Doppler variables.

Maternal demographic and clinical variables included age, gravidity, parity, number of previous cesarean deliveries, body mass index, PIH subtype, and laboratory parameters. Laboratory variables comprised serum creatinine, aspartate aminotransferase, alanine aminotransferase, and platelet count. Obstetric and neonatal variables included gestational age (GA) at ultrasound (US) assessment, GA at delivery, mode of delivery, indication for delivery, neonatal sex, birth weight, 1 and 5 min Apgar scores, and NICU admission.

### 2.4. Data Sources and Measurements

Maternal demographic and clinical data were prospectively recorded on the study case report form. Laboratory parameters from maternal blood samples obtained closest to delivery and perinatal outcome data were retrieved from the hospital database after delivery. Birth weight percentiles were calculated according to the INTERGROWTH-21st newborn size standards, and neonates with a birth weight below the 10th percentile were classified as small for gestational age (SGA) [19]. Ultrasonographic and Doppler examinations were performed on an ARIETTA 850 system (Hitachi Medical Corporation, Tokyo, Japan) equipped with a 3.5 MHz convex transducer. During follow-up, Doppler assessments were repeated weekly, and for each case, the last measurement before delivery—the most recent assessment obtained within 7 days before birth—was included in the analyses. GA at US was defined as the gestational age at this last Doppler assessment included in the analyses.

Fetal Doppler examinations, including UA PI, MCA PI, UtA PI, and DV Doppler measurements, were performed in accordance with the guidelines of the International Society of Ultrasound in Obstetrics and Gynecology (ISUOG), using pulsed-wave Doppler during fetal quiescence [20]. UA PI was calculated from technically adequate consecutive waveforms obtained from a free loop of the umbilical cord. MCA PI was measured from the proximal segment of the vessel after color Doppler visualization of the circle of Willis in the transthalamic plane, with the angle of insonation kept as close to 0° as possible. Right and left UtA PI were obtained transabdominally distal to the external iliac artery crossover, and their mean was calculated. As part of the exclusion screening, DV flow was first identified with color Doppler, and waveforms were obtained in a fetal abdominal midsagittal plane with a narrow sample gate and low wall filter. CPR was derived by dividing MCA PI by UA PI, and the resulting values were assigned percentiles according to the Fetal Medicine Foundation (FMF) reference charts [21]. CPUR was calculated by dividing CPR by mean UtA PI.

Maternal RA Doppler assessment was performed transabdominally with the patient in the lateral decubitus position. Because the enlarged uterus in late gestation may hinder visualization of the left kidney and left RA, measurements were standardized using the right RA. After color Doppler demonstration of right RA flow, pulsed-wave sampling was performed at the interlobar artery along the border of the medullary pyramids. Throughout this study, the term ‘right RA PI’ denotes the pulsatility index recorded at this interlobar level. During spectral Doppler recording, the sample gate was set at 2 mm, the wall filter at 100 Hz, and the angle of insonation at <60°. Measurements were obtained from at least three uniform consecutive Doppler waveforms during transient maternal breath-holding. Right RA PI was automatically calculated by the ultrasound system from the recorded waveforms. CPRR, as defined in this study, was formulated by dividing CPR by right RA PI.

### 2.5. Bias

Selection bias was addressed through consecutive recruitment of all eligible pregnant women and uniform application of the prespecified eligibility criteria. Measurement consistency was enhanced by assigning every Doppler examination to the same experienced investigator (YK), who used one US system and transducer and followed a uniform acquisition protocol. Regarding outcome ascertainment, non-reassuring fetal status was determined by cardiotocographic assessment according to standard clinical criteria. The study-derived indices evaluated in the present analysis, including CPR, CPUR, and CPRR, were not used to guide decisions regarding the timing or mode of delivery. To reduce confounding bias when evaluating the association between CPRR and APO, clinically relevant potential confounding variables were included in the multivariable logistic regression models.

### 2.6. Study Size

Sample size was calculated a priori using G*Power version 3.1.9.4 [22]. The effect size was set at 0.708 based on the CPUR parameter reported by Karabay et al. [23]. For a two-tailed independent-samples t test, assuming α = 0.05, power = 0.80, and an allocation ratio (n2/n1) of 1.44, the minimum required sample size was 68 cases in total (28 and 40, respectively). The final cohort included 37 patients with APO and 68 patients without APO, thereby exceeding the a priori minimum. Because CPRR was a newly defined index without prior data at the design stage, the closest available comparator, CPUR, was used for this calculation. We acknowledge that this a priori estimate was pragmatic and indirect and does not directly correspond to the primary ROC or multivariable logistic regression analyses.

### 2.7. Statistical Analysis

Statistical analyses were conducted using SPSS 26.0 (IBM Corp., Armonk, NY, USA). The primary analyses focused on the association between CPRR and APO and the discriminative performance of CPRR for APO. Comparisons with CPR and CPUR were considered secondary comparative analyses, whereas subgroup and sensitivity analyses were interpreted as exploratory. Continuous variables were tested for normality with the Shapiro–Wilk test. Data are expressed as mean ± standard deviation, median (minimum–maximum), or number (%), as appropriate. Continuous variables were compared using Student’s *t*-test, Welch’s *t*-test, or the Mann–Whitney U test, whereas categorical variables were analyzed using the chi-square or Fisher’s exact test. For multicategory variables, Bonferroni-corrected post hoc analysis based on adjusted standardized residuals was performed to identify the source of within-variable group differences. Spearman correlation analysis was used to assess the relationships among CPR, right RA PI, and CPRR. The discriminative ability of CPR, CPUR, and CPRR for APO was evaluated using receiver operating characteristic (ROC) analysis. All 105 analyzed pregnancies had complete Doppler measurements and APO status; therefore, no case was excluded from the ROC analyses because of missing data and no imputation was required. The area under the curve (AUC) and its 95% confidence interval (CI) were estimated using the standard nonparametric ROC procedure, with tied predictor values handled within this analysis. The optimal cutoff was defined as the value maximizing the Youden index, calculated as sensitivity + specificity − 1, and was reported with apparent sensitivity and specificity as derivation-cohort estimates. Pairwise comparisons of correlated ROC curves for CPR, CPUR, and CPRR were performed using DeLong’s test in Python (version 3.13.5). Internal validation of ROC performance was performed using 1000 bootstrap resamples to estimate optimism-corrected AUC values for each Doppler index in Python. Stratified analyses according to diagnosis group were performed for CPRR using Mann–Whitney U testing and ROC analysis and were interpreted descriptively because of limited subgroup event counts. Univariable and multivariable binary logistic regression analyses were performed to evaluate factors associated with APO, and the results were presented as crude odds ratio (OR) and adjusted odds ratio (aOR), respectively, along with 95% CI. To avoid multicollinearity and overfitting, CPR, CPUR, and CPRR were entered into separate multivariable models. Adjustment variables were prespecified; each primary model included GA at US assessment, diagnosis group, and one Doppler index. The role of birth-weight-based SGA status was additionally explored in a supplementary multivariable model including CPRR, GA at US assessment, diagnosis group, and SGA status. Exploratory analyses according to birth-weight-based SGA status included comparisons of Doppler indices between SGA and non-SGA pregnancies and, within the non-SGA subgroup, comparisons of CPR, CPUR, and CPRR between cases with and without APO using distribution-appropriate group comparison tests. Discrimination for APO within the non-SGA subgroup was evaluated using ROC analysis. Model calibration, collinearity among predictors, and explanatory performance were examined using the Hosmer–Lemeshow test, variance inflation factor (VIF), and Nagelkerke R^2^, respectively. The Akaike information criterion (AIC) was calculated to compare the balance between model fit and parsimony across models. Statistical significance was set at *p* < 0.05.

## 3. Results

**Figure 1:**
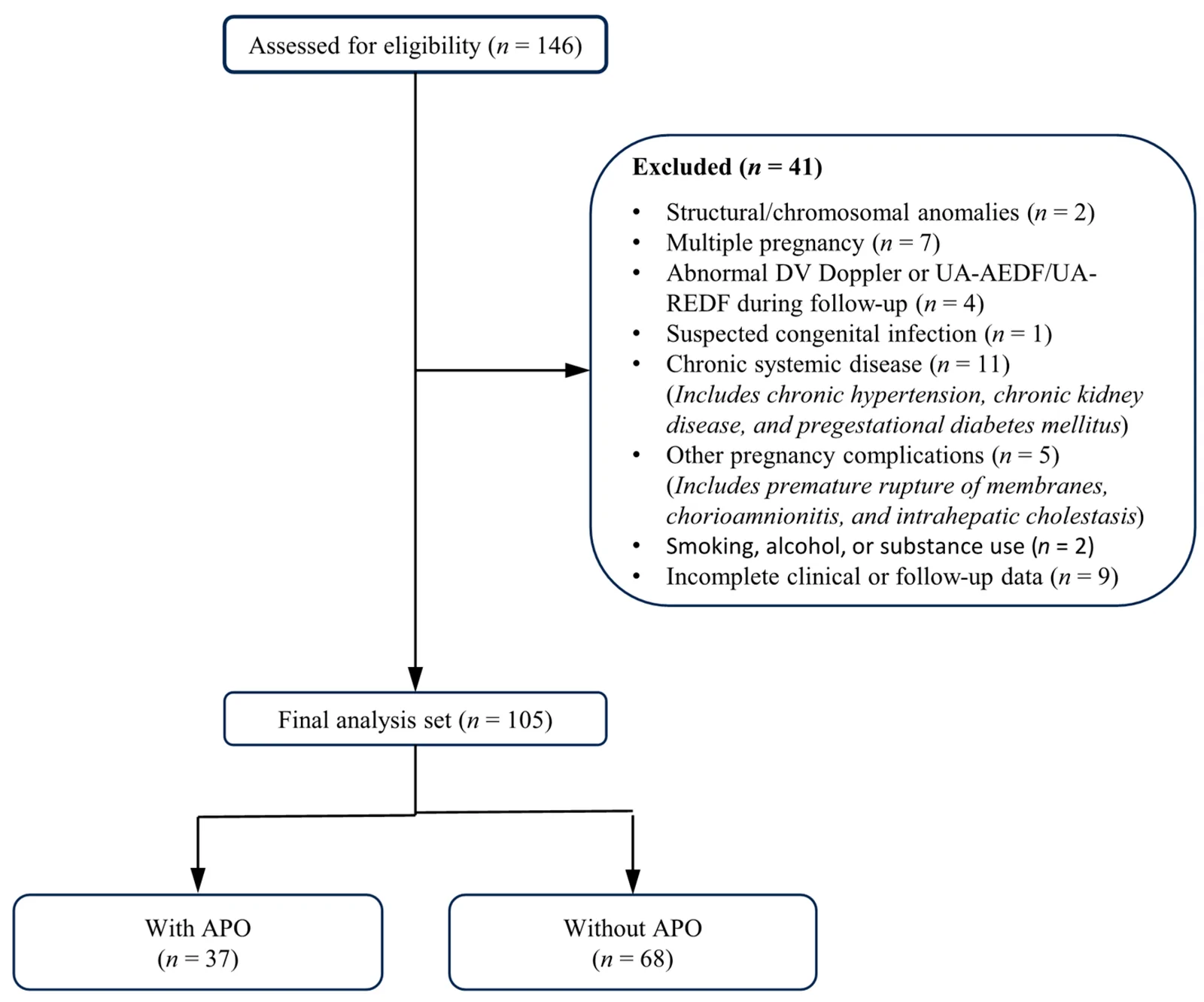
Flow chart of participant selection and exclusion reasons. Abbreviations: APO, adverse perinatal outcome; DV, ductus venosus; UA-AEDF, umbilical artery absent end-diastolic flow; UA-REDF, umbilical artery reversed end-diastolic flow.

During the study period, 146 pregnant women were assessed for eligibility; of these, 41 were excluded, leaving 105 in the final analysis (Figure 1). Of these cases, 37 (35.2%) constituted the with APO group, whereas 68 (64.8%) comprised the without APO group. The median interval between Doppler examination and delivery was 4 days (range 0–7); between laboratory testing and delivery, 3 days (range 0–7); and between laboratory testing and Doppler examination, 1 day (range 0–4).

The demographic, clinical, and delivery characteristics of the study cohort are presented in Table 1. Maternal demographics, obstetric history, laboratory parameters, GA at US and delivery, and mode of delivery were comparable between the groups. The frequency of preeclampsia was higher in the APO group (86.5% vs. 45.6%; *p* < 0.001), whereas gestational hypertension was less frequent (13.5% vs. 54.4%; *p* < 0.001). Birth weight was lower in the APO group (2210 g vs. 2682 g; *p* = 0.005). No neonatal deaths occurred in the cohort; therefore, although neonatal death was included as a predefined component of APO, it contributed no events to the endpoint. SGA, defined as birth weight below the 10th percentile, was more frequent in the APO group than in the non-APO group (51.4% vs. 27.9%; *p* = 0.017). Within the preeclampsia subgroup, birth-weight-based SGA was present in 28 of 63 cases (44.4%) and was numerically more frequent among APO-positive than APO-negative cases (56.3% vs. 32.3%; *p* = 0.055). In exploratory analyses according to SGA status, SGA pregnancies had lower CPR (1.37 ± 0.23 vs. 1.49 ± 0.23; *p* = 0.013), lower CPUR (1.30 ± 0.32 vs. 1.51 ± 0.29; *p* = 0.001), lower CPRR (1.35 ± 0.27 vs. 1.50 ± 0.35; *p* = 0.022), and higher UA PI (1.00 ± 0.12 vs. 0.95 ± 0.12; *p* = 0.045) than non-SGA pregnancies, whereas MCA PI, mean UtA PI, and right RA PI did not differ significantly. Among non-SGA pregnancies (*n* = 67), CPRR remained significantly lower in APO-positive than APO-negative cases (1.27 ± 0.25 vs. 1.59 ± 0.34; *p* = 0.001) and showed apparent discrimination for APO (AUC 0.784; 95% CI 0.662–0.906; *p* < 0.001), whereas CPR and CPUR did not significantly discriminate APO in this subgroup.

Doppler parameters and derived Doppler indices are shown in Table 2. UA PI was higher in the APO group (1.00 ± 0.12 vs. 0.95 ± 0.12; *p* = 0.041). No significant between-group differences were observed in MCA PI, CPR, or CPR percentile. Mean UtA PI and right RA PI were higher in the APO group (*p* = 0.034 and *p* = 0.002, respectively). CPUR was lower in the APO group (1.30 ± 0.35 vs. 1.50 ± 0.27; *p* = 0.002). CPRR was also lower in the APO group (1.24 ± 0.21 vs. 1.55 ± 0.32; *p* < 0.001).

In the overall cohort, CPRR showed a weak positive correlation with CPR (ρ = 0.258; *p* = 0.008) and a strong inverse correlation with right RA PI (ρ = −0.738; *p* < 0.001), whereas CPR and right RA PI were moderately correlated (ρ = 0.410; *p* < 0.001).

Doppler indices and perinatal outcomes were compared between the preeclampsia (*n* = 63) and gestational hypertension (*n* = 42) subgroups (Table 3). Compared with the gestational hypertension group, the preeclampsia group had higher median UA PI (1.05 vs. 0.86; *p* < 0.001), and right RA PI did not differ significantly (*p* = 0.878). In contrast, CPR (1.34 ± 0.18 vs. 1.58 ± 0.23; *p* < 0.001), CPR percentile (5 vs. 15; *p* < 0.001), CPUR (1.29 ± 0.29 vs. 1.63 ± 0.23; *p* < 0.001), and CPRR (1.28 [0.85–2.00] vs. 1.53 [1.01–2.41]; *p* < 0.001) were significantly lower in the preeclampsia group. MCA PI and mean UtA PI were comparable between the subgroups. Women with preeclampsia delivered earlier than those with gestational hypertension (*p* = 0.028), whereas birth weight did not differ significantly between the subgroups (*p* = 0.079). NICU admission occurred only in the preeclampsia subgroup (28.6% vs. 0%; *p* < 0.001). In a stratified analysis by diagnosis group, CPRR remained significantly lower in APO-positive than APO-negative cases within the preeclampsia subgroup (median 1.21 vs. 1.48; *p* < 0.001), with an apparent AUC of 0.781 (95% CI 0.663–0.898; *p* < 0.001). In the gestational hypertension subgroup, CPRR did not significantly discriminate APO (AUC 0.565; 95% CI 0.329–0.801; *p* = 0.641), although interpretation was limited by the small number of APO events (*n* = 5).

**Figure 2:**
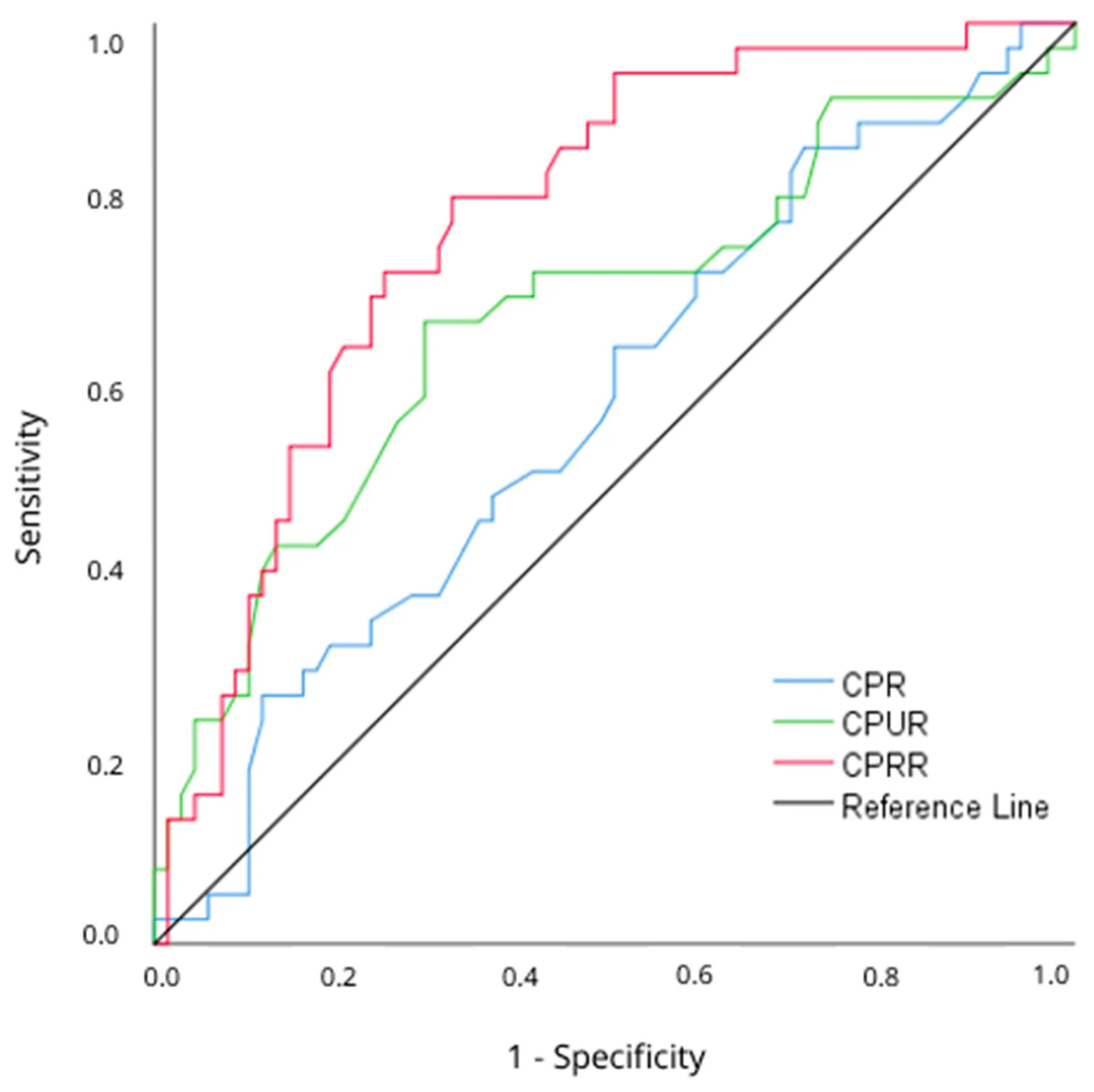
ROC curves of CPR, CPUR, and CPRR for predicting adverse perinatal outcome. CPRR showed an AUC of 0.786, compared with 0.683 for CPUR and 0.584 for CPR. The diagonal line represents the reference line indicating no discriminative ability. Abbreviations: AUC, area under the receiver operating characteristic curve; CPR, cerebroplacental ratio; CPRR, cerebroplacental–renal ratio; CPUR, cerebroplacental–uterine ratio; ROC, receiver operating characteristic.

In the ROC analysis performed to evaluate discriminative performance for APO, the AUC values were 0.584 for CPR (95% CI 0.470–0.697; *p* = 0.158), 0.683 for CPUR (95% CI 0.571–0.796; *p* = 0.002), and 0.786 for CPRR (95% CI 0.698–0.875; *p* < 0.001) (Table 4, Panel A; Figure 2). Pairwise DeLong comparisons showed that the AUC of CPRR was significantly higher than that of CPR (ΔAUC = 0.203; 95% CI 0.082–0.324; z = 3.28; *p* = 0.001) and CPUR (ΔAUC = 0.103; 95% CI 0.002–0.204; z = 2.00; *p* = 0.046), whereas the difference between CPUR and CPR was not statistically significant (ΔAUC = 0.100; 95% CI −0.016 to 0.215; z = 1.69; *p* = 0.091) (Table 4, Panel B). Bootstrap internal validation using 1000 resamples showed no meaningful optimism for CPRR (optimism <0.001), with an apparent AUC of 0.786 and an optimism-corrected AUC of 0.787. The optimism-corrected AUC values were 0.682 for CPUR and 0.572 for CPR. The Youden-derived cutoff for CPRR was 1.40, yielding a sensitivity of 81.1% and specificity of 67.6% in the derivation cohort.

**Figure 3:**
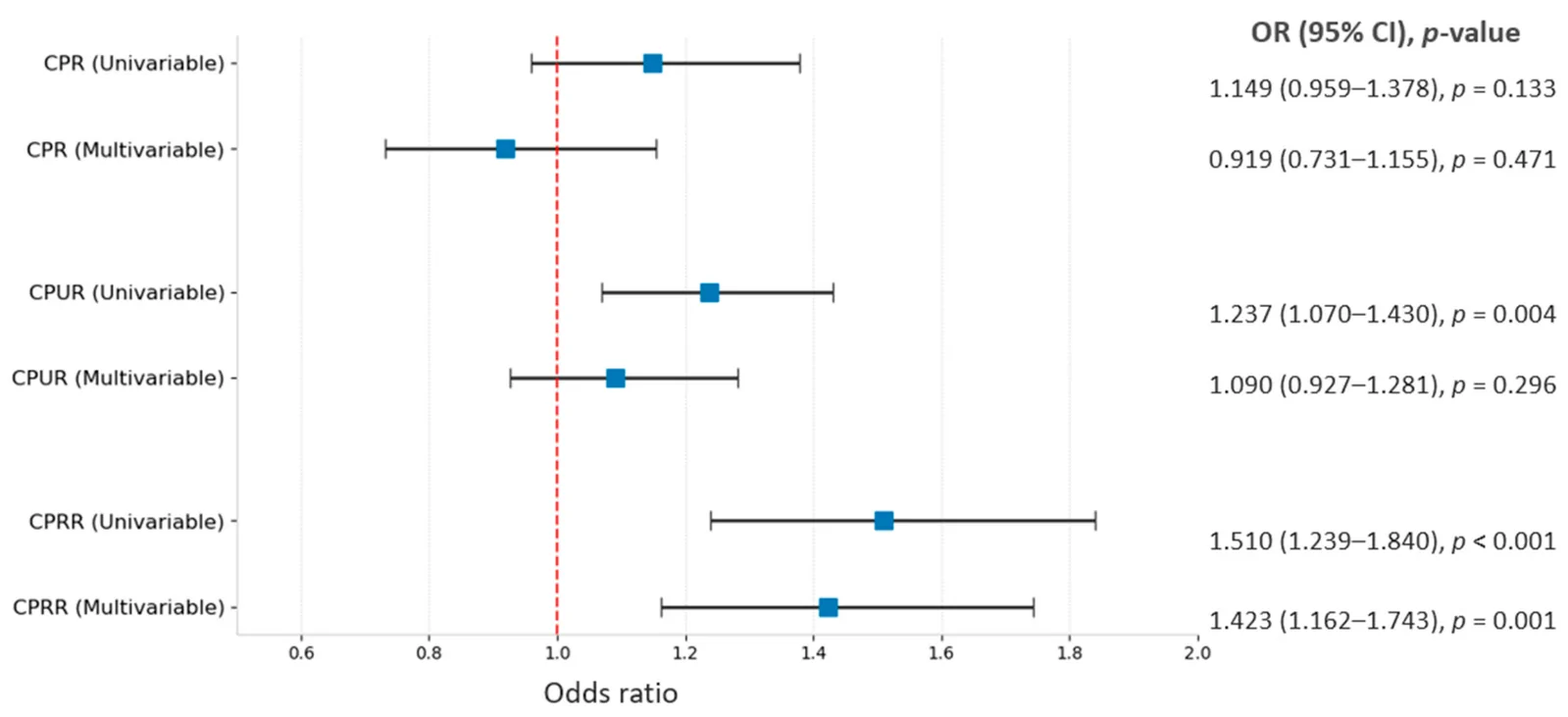
Forest plot of univariable and multivariable odds ratios for CPR, CPUR, and CPRR in predicting adverse perinatal outcome. Squares indicate odds ratios, and horizontal error bars represent 95% CI. Multivariable models were adjusted for gestational age at ultrasound assessment and diagnosis group. Odds ratios for CPR, CPUR, and CPRR refer to each 0.1-unit decrease. The vertical dashed red line indicates the line of no effect (OR = 1.0). Abbreviations: CI, confidence interval; CPR, cerebroplacental ratio; CPRR, cerebroplacental–renal ratio; CPUR, cerebroplacental–uterine ratio; OR, odds ratio.

Univariable and multivariable logistic regression results are summarized in Table 5 and Figure 3. In the univariable analysis, UA PI, mean UtA PI, right RA PI, CPUR, CPRR, and preeclampsia were associated with APO, whereas GA at US assessment and CPR were not. In the multivariable analysis, each Doppler index was entered into a separate model adjusted for GA and diagnosis group. Following multivariable adjustment, neither CPR (aOR 0.919; 95% CI 0.731–1.155; *p* = 0.471) nor CPUR (aOR 1.090; 95% CI 0.927–1.281; *p* = 0.296) remained statistically significant. CPRR remained independently associated with APO (aOR 1.423 per 0.1-unit decrease; 95% CI 1.162–1.743; *p* = 0.001); in the same model, preeclampsia also remained significant (aOR 4.518; *p* = 0.010). Among the compared prespecified models, the CPRR-based model had a higher Nagelkerke R^2^ (0.377) and a lower AIC (110.647) than the CPR- and CPUR-based models. An exploratory model additionally including SGA status, together with CPRR, GA at US assessment, and diagnosis group, showed that CPRR remained independently associated with APO (adjusted OR 1.410 per 0.1-unit decrease; 95% CI 1.148–1.732; *p* = 0.001). All three models demonstrated acceptable calibration according to the Hosmer–Lemeshow test (*p* > 0.05), and no multicollinearity was detected (all VIF < 2.5).

## 4. Discussion

In this prospective cohort, we examined whether the newly defined CPRR was associated with APO in PIH and compared its discriminative performance with that of CPR and CPUR. Among cases that developed APO, maternal right RA PI and mean UtA PI were higher, whereas CPUR and CPRR were lower. In ROC analysis, CPRR showed higher apparent discrimination than CPUR and CPR, and this difference was supported by pairwise DeLong comparisons. After adjustment for GA and diagnosis group, CPR and CPUR lost statistical significance, whereas CPRR remained the only Doppler index independently associated with APO (aOR 1.423 per 0.1-unit decrease; *p* = 0.001). The CPRR-based model also had more favorable model-fit indices than the CPR- and CPUR-based models. In addition, subgroup analyses showed lower CPR, CPUR, and CPRR values in pregnancies with preeclampsia than in those with gestational hypertension, whereas right RA PI was comparable.

Among the evaluated Doppler indices, CPRR retained an independent association with APO and showed greater discriminative performance than CPR and CPUR within this cohort, a pattern that may reflect its integration of fetal cerebroplacental adaptation and maternal renal vascular resistance. This formulation was chosen because lower CPR and higher right RA PI represent unfavorable fetal and maternal hemodynamic directions, respectively; therefore, lower CPRR values reflect the coexistence of these two unfavorable features. Consistent with this formulation, CPRR showed only a weak positive correlation with CPR but a strong inverse correlation with right RA PI, suggesting that the renal Doppler component substantially influences the behavior of the index. Nevertheless, because CPRR is mathematically derived from CPR and right RA PI, this correlation pattern should be interpreted descriptively rather than as proof of an independent physiological mechanism. The ROC-based difference in favor of CPRR was further supported by pairwise DeLong comparisons, which showed significantly higher AUCs for CPRR than for both CPR and CPUR. Together, these observations raise the possibility that CPRR captured a more consistent integrated hemodynamic signal than CPR and CPUR in this selected PIH population. Because the present analysis was a single-cohort model-development study, the proposed cutoff should be regarded as exploratory and requires confirmation in independent cohorts before it can be applied as a clinical threshold. Furthermore, the stratified analysis showed that CPRR remained lower in APO-positive cases within the preeclampsia subgroup, suggesting that the observed association was not solely driven by the preeclampsia–gestational hypertension distinction; however, residual disease-severity-related confounding remains possible. Overall, these results should be interpreted as evidence of an associative statistical relationship and cohort-specific discriminative performance rather than proof of causality, clinically meaningful incremental predictive value, clinical superiority over established indices, or a validated clinical marker for APO.

CPR is a well-recognized fetal Doppler marker of fetal blood flow redistribution and placental insufficiency. Low CPR values have been associated with the risk of stillbirth and perinatal loss, as well as with adverse perinatal outcomes in hypertensive pregnancies [7,24]. In contrast, CPR’s lack of significant discrimination for APO and failure to remain independent suggest that assessment of fetoplacental circulation alone may be limited in this patient group. The fact that measurements were performed close to delivery and at ≥34 weeks of gestation, together with the inclusion of only PIH cases, may partly explain this observation; this result reflects the limitation of using CPR alone in this context rather than questioning its overall clinical value. Indeed, although CPR has shown high diagnostic accuracy for predicting perinatal outcomes in some studies, other reports have found its discriminative ability to be more limited or have failed to demonstrate a significant association with adverse neonatal outcomes [8,9,23].

CPUR evaluates fetoplacental and uteroplacental circulation by adding mean UtA PI information to CPR. In PIH cohorts, evidence supports its potential to predict perinatal outcomes [11,23,25]. Consistent with these data, CPUR showed a higher AUC than CPR in the ROC analysis and was associated with APO in the univariable analysis. However, the loss of CPUR independence after adjustment indicates that, although the uterine artery component may add information, it may not sufficiently capture APO-associated hemodynamic variability on its own. This finding also appears consistent with studies reporting that, even in low-risk term pregnancies, adding uterine Doppler information to CPR in late pregnancy may not always significantly improve the prediction of perinatal outcomes [26,27].

PIH, and particularly preeclampsia, is regarded as a disease spectrum that begins with placental processes but is associated with systemic endothelial dysfunction and renal microvascular involvement [6,13]. Maternal renal hemodynamics may therefore provide complementary information from a vascular compartment distinct from the uteroplacental circulation. Previous studies suggest that maternal renal Doppler parameters may vary in these cases and may reflect vascular impedance [28]. However, the literature is not entirely consistent regarding the clinical role of renal Doppler parameters; a meta-analysis reported that RA resistive index and PI did not differ significantly between preeclamptic and healthy pregnancies, that significant associations were mainly observed for venous parameters, and that the role of these indices in clinical practice remains unclear [16]. In this context, the higher right RA PI observed in the APO group may offer a biologically plausible explanation for the more consistent association of CPRR compared with CPR and CPUR. These data position renal Doppler measurements not as direct indicators of PIH severity but as complementary signals of the systemic vascular component of the disease.

A notable finding in the multivariable analysis was that CPRR retained an independent association with APO despite the persistence of preeclampsia as a significant predictor in the same model. Although this pattern may suggest that CPRR provides hemodynamic information complementary to diagnosis group, it should be viewed as a possible hemodynamic contribution rather than a direct explanation of preeclampsia-related risk or a stand-alone representation of the disease process. Because multiple Doppler indices and separate multivariable models were evaluated, the findings should not be overinterpreted, and uncertainty from multiple analyses should be considered. Thus, the independent association observed for CPRR in this cohort does not establish it as a validated tool that can be used alone in clinical decision-making; rather, it suggests that CPRR may be considered a complementary investigational parameter that jointly assesses fetoplacental and maternal renal hemodynamic information.

The relationship between CPRR and fetal growth warrants specific consideration. CPRR was lower in SGA than in non-SGA pregnancies, indicating sensitivity to growth-related hemodynamic changes. Importantly, however, CPRR remained significantly lower among APO-positive cases within the birth-weight-based non-SGA subgroup, whereas CPR and CPUR did not significantly discriminate APO in this subgroup. In the exploratory multivariable model including SGA status, CPRR remained independently associated with APO, whereas SGA status itself was not statistically significant. These findings suggest that the association between CPRR and APO may not be confined to pregnancies with birth-weight-based SGA. They should nonetheless be interpreted cautiously, as the analyses were exploratory and SGA was defined postnatally; residual confounding by unmeasured antenatal growth restriction cannot be excluded, and the independent relationship between CPRR, fetal growth status, and APO cannot be definitively established from the present data.

This study has several strengths. Its prospective design, consecutive enrollment of eligible pregnant women, and predefined eligibility criteria helped limit selection bias. The use of the same US system, a standardized protocol, and a single experienced investigator for all Doppler measurements improved internal consistency. Using the last Doppler assessment before delivery, with all measurements within 7 days before birth, allowed for evaluation of the temporal relationship between antenatal hemodynamics and perinatal outcomes within a standardized interval. In addition, focusing exclusively on PIH enabled a comparative assessment of CPR, CPUR, and the newly defined CPRR within a high-risk, well-defined disease spectrum. Evaluating each Doppler index in a separate adjusted model, with assessment of model fit and multicollinearity, strengthened the analytical approach. To our knowledge, this is among the first studies to evaluate CPRR, which adds a maternal renal Doppler component to fetoplacental hemodynamic information, in the context of APO among cases of PIH.

However, several limitations of this study should be considered. First, the single tertiary-center setting may have increased the representation of severe or complex PIH cases, limiting generalizability to broader obstetric populations. In addition, the relatively limited sample size may have affected statistical power, particularly in subgroup analyses. In particular, because only 37 APO events occurred, the multivariable regression estimates remain susceptible to overfitting, model instability, and imprecision; although the primary models were deliberately restricted to a small number of prespecified predictors, these concerns cannot be entirely eliminated in a cohort of this size. Although Bonferroni-corrected post hoc testing was applied for multicategory variables, no global multiplicity correction was applied across all non-primary analyses; therefore, non-primary findings should be interpreted cautiously because of the potential for type I error inflation. The APO endpoint was mainly driven by non-reassuring fetal status and NICU admission, both of which may be influenced by clinical decision-making, institutional practice, and GA. However, non-reassuring fetal status was determined by cardiotocography, and the study-derived Doppler indices were not used to guide delivery management; a sensitivity analysis excluding non-reassuring fetal status yielded results consistent with the primary analysis. Nevertheless, residual ascertainment and management-related bias cannot be fully excluded. A further limitation is that quantitative blood pressure and proteinuria values, detailed disease-severity markers, antenatal fetal biometry (estimated fetal weight and abdominal circumference percentiles), and antenatal fetal growth-restriction status were not recorded as separate variables for modeling. The absence of standardized antenatal fetal biometry and antenatal fetal growth-restriction status therefore precluded formal antenatal growth-restriction phenotyping. Although adjustment for diagnosis group partly captures the underlying disease phenotype, unmeasured confounding may persist. Birth-weight-based SGA status was reported descriptively and additionally examined in an exploratory model; however, as a postnatal measure, it cannot fully substitute for antenatal growth-restriction phenotyping. Doppler assessment in this study was restricted to ≥34 weeks’ gestation, and the analyzed measurement for each case was obtained within 7 days before birth. Because cerebroplacental and maternal renal Doppler indices may vary with GA, both the observed associations and the proposed CPRR cutoff may not be transferable to earlier gestations or screening settings and must be evaluated separately before being applied in those contexts. Another important limitation is that maternal renal Doppler assessment was performed only using right RA PI by a single experienced investigator for technical standardization. Although this approach may improve measurement consistency and feasibility in late pregnancy, it did not allow for bilateral renal hemodynamic evaluation, and formal intraobserver and interobserver reproducibility was not assessed.

Because the present study did not include uncomplicated pregnancies, CPRR values in physiologically normal pregnancies could not be characterized; therefore, future studies should include such pregnancies to define GA-specific CPRR reference ranges and improve interpretability. Formal reproducibility assessment should be incorporated into subsequent studies. Although bootstrap internal validation indicated no meaningful optimism in the AUC estimate for CPRR, this internal procedure does not confirm calibration or external validity. Ultimately, before CPRR or the proposed cutoff can be translated into clinical use, they should be validated in larger, multicenter prospective studies including different gestational ages and independent validation cohorts. Within such studies, standardized antenatal fetal biometry, including estimated fetal weight and abdominal circumference percentiles, and formal antenatal fetal growth-restriction classification should be incorporated. Adequately powered multivariable models should then be used to address residual confounding and to determine whether CPRR provides incremental risk information beyond fetal growth status, established Doppler indices, and clinical variables.

## 5. Conclusions

In this prospective cohort study, CPRR, which integrates fetal cerebroplacental adaptation with maternal renal Doppler information, was independently associated with APO in pregnancies diagnosed with PIH, with higher apparent discrimination than CPR and CPUR in this cohort. Although these findings suggest that CPRR may be a noteworthy hemodynamic marker in this hypertensive pregnancy cohort, they should not be interpreted as indicating that the index is superior or sufficient to guide patient management. Translation of these results into a clinical tool suitable for routine use will require comprehensive validation through independent, multicenter prospective studies.

## Figures and Tables

**Table 1:** Baseline demographic, clinical, and delivery characteristics of the study cohort according to the occurrence of adverse perinatal outcome.

Variable	Total Cohort (*n* = 105)	With APO (*n* = 37)	Without APO (*n* = 68)	*p*
Maternal demographic characteristics				
Maternal age (years)	33 (18–47)	34 (18–46)	33 (19–47)	0.846
BMI (kg/m^2^)	33.8 ± 5.3	34.6 ± 5.3	33.4 ± 5.2	0.240
Gravidity	4 (1–13)	4 (1–10)	4 (1–13)	0.224
Parity	1 (0–8)	1 (0–8)	1 (0–8)	0.747
Nulliparity	39 (37.1)	12 (32.4)	27 (39.7)	0.461
Number of previous cesareans	0 (0–5)	0 (0–4)	0 (0–5)	0.052
Clinical characteristics at US assessment				
Preeclampsia	63 (60.0)	32 (86.5)	31 (45.6)	**<0.001**
Gestational hypertension	42 (40.0)	5 (13.5)	37 (54.4)	
GA at US assessment (weeks)	36.0 (34.0–38.3)	35.8 (34.0–37.6)	36.0 (34.1–38.3)	0.322
Laboratory parameters				
Serum creatinine (mg/dL)	0.71 ± 0.16	0.70 ± 0.18	0.71 ± 0.15	0.738
Aspartate aminotransferase (U/L)	33.7 ± 10.3	33.8 ± 10.8	33.6 ± 10.1	0.892
Alanine aminotransferase (U/L)	32.3 ± 9.5	33.1 ± 10.4	31.8 ± 8.9	0.494
Platelet (10^3^/µL)	243.4 ± 75.3	233.6 ± 78.5	248.7 ± 73.5	0.328
Delivery characteristics				
GA at delivery (weeks)	36.7 (34.1–39.0)	36.2 (34.1–37.9)	36.8 (34.4–39.0)	0.317
Mode of delivery				0.946
Vaginal delivery	15 (14.3)	5 (13.5)	10 (14.7)	
Elective cesarean section	27 (25.7)	9 (24.3)	18 (26.5)	
Non-elective cesarean section	63 (60.0)	23 (62.2)	40 (58.8)	
Indication for delivery				**<0.001**
Non-reassuring fetal status ^a^	19 (18.1)	19 (51.4)	0 (0)	
Reached planned GA	29 (27.6)	9 (24.3)	20 (29.4)	
Severe preeclampsia, HELLP syndrome or other ^a^	57 (54.3)	9 (24.3)	48 (70.6)	
Perinatal outcome				
Birth weight (g)	2505 (1280–4310)	2210 (1280–3985)	2682 (1700–4310)	**0.005**
SGA (Birth weight < 10 percentile)	38 (36.2)	19 (51.4)	19 (27.9)	**0.017**
Male sex	53 (50.5)	17 (45.9)	36 (52.9)	0.493
Apgar score at 1 min	7 (3–9)	6 (3–9)	7 (5–9)	**0.009**
Apgar score at 5 min	9 (5–10)	9 (5–10)	9 (8–10)	0.319
Apgar score < 7 at 5 min	3 (2.9)	3 (8.1)	0 (0)	**0.041**
NICU admission	18 (17.1)	18 (48.6)	0 (0)	**<0.001**

Data are presented as mean ± standard deviation, median (minimum–maximum), or number (percentage), as appropriate. Bold values indicate statistical significance (*p* < 0.05). ^a^ Denotes a significant between-group difference within the indication for delivery category based on Bonferroni-corrected post hoc adjusted standardized residual analysis. Indication for delivery should be interpreted descriptively because non-reassuring fetal status was included as a component of APO. No cases of abnormal amniotic fluid volume or neonatal death were observed in the study cohort. Abbreviations: APO, adverse perinatal outcome; BMI, body mass index; GA, gestational age; HELLP, hemolysis, elevated liver enzymes, and low platelet count; NICU, neonatal intensive care unit; US, ultrasound.

**Table 2:** Doppler parameters and derived Doppler indices according to adverse perinatal outcome status.

Variable	With APO (*n* = 37)	Without APO (*n* = 68)	*p*
UA PI	1.00 ± 0.12	0.95 ± 0.12	**0.041**
MCA PI	1.38 ± 0.16	1.38 ± 0.23	0.917
CPR	1.39 ± 0.22	1.46 ± 0.23	0.131
CPR percentile	6 (1–49)	9 (1–57)	0.128
Mean UtA PI	1.07 (0.77–1.70)	0.98 (0.52–1.64)	**0.034**
Right RA PI	1.14 (0.78–1.45)	0.92 (0.69–1.44)	**0.002**
CPUR	1.30 ± 0.35	1.50 ± 0.27	**0.002**
CPRR	1.24 ± 0.21	1.55 ± 0.32	**<0.001**

Data are presented as mean ± standard deviation or median (minimum–maximum), as appropriate. Bold values indicate statistical significance (*p* < 0.05). Abbreviations: APO, adverse perinatal outcome; CPR, cerebroplacental ratio; CPRR, cerebroplacental–renal ratio; CPUR, cerebroplacental–uterine ratio; MCA, middle cerebral artery; PI, pulsatility index; RA, renal artery; UA, umbilical artery; UtA, uterine artery.

**Table 3:** Comparison of Doppler indices and perinatal outcomes between the preeclampsia and gestational hypertension subgroups.

Variable	Preeclampsia (*n* = 63)	GHT (*n* = 42)	*p*
GA at US assessment (weeks)	36.0 (34.0–37.7)	36.1 (34.1–38.3)	0.056
UA PI	1.05 (0.69–1.32)	0.86 (0.67–1.28)	**<0.001**
MCA PI	1.40 (0.93–1.73)	1.34 (0.99–2.15)	0.799
CPR	1.34 ± 0.18	1.58 ± 0.23	**<0.001**
CPR percentile	5 (1–47)	15 (1–57)	**<0.001**
Mean UtA PI	1.04 (0.64–1.70)	0.97 (0.52–1.63)	0.056
Right RA PI	1.03 (0.69–1.45)	1.06 (0.69–1.44)	0.878
CPUR	1.29 ± 0.29	1.63 ± 0.23	**<0.001**
CPRR	1.28 (0.85–2.00)	1.53 (1.01–2.41)	**<0.001**
GA at delivery (weeks)	36.5 (34.1–37.9)	36.8 (34.9–39.0)	**0.028**
Birth weight (g)	2335 (1280–4240)	2705 (1750–4310)	0.079
Apgar score < 7 at 5 min	3 (4.8)	0 (0)	0.273
NICU admission	18 (28.6)	0 (0)	**<0.001**

Data are presented as mean ± standard deviation, median (minimum–maximum), or number (percentage), as appropriate. Statistically significant values (*p* < 0.05) are highlighted in bold. Abbreviations: CPR, cerebroplacental ratio; CPRR, cerebroplacental–renal ratio; CPUR, cerebroplacental–uterine ratio; GA, gestational age; GHT, gestational hypertension; MCA, middle cerebral artery; NICU, neonatal intensive care unit; PI, pulsatility index; RA, renal artery; UA, umbilical artery; US, ultrasound; UtA, uterine artery.

**Table 4:** Receiver operating characteristic analysis and pairwise DeLong comparisons of CPR, CPUR, and CPRR for predicting adverse perinatal outcome.

(**A**) Receiver operating characteristic analysis.						
**Variable**	**Cutoff**	**AUC**	**95% CI**	**Sensitivity (%)**	**Specificity (%)**	***p***
CPR	1.56	0.584	0.470–0.697	86.5	29.4	0.158
CPUR	1.37	0.683	0.571–0.796	67.6	70.6	**0.002**
CPRR	1.40	0.786	0.698–0.875	81.1	67.6	**<0.001**
(**B**) Pairwise DeLong comparisons of correlated ROC curves.						
**Comparison**	**ΔAUC**	**SE**	**95% CI**	**z Statistic**	***p***	
CPRR versus CPR	0.203	0.062	0.082–0.324	3.28	**0.001**	
CPRR versus CPUR	0.103	0.051	0.002–0.204	2.00	**0.046**	
CPUR versus CPR	0.100	0.059	−0.016 to 0.215	1.69	0.091	

Panel A presents the area under the receiver operating characteristic curve for each index, with the Youden-derived optimal cutoff, sensitivity, and specificity. Panel B presents pairwise comparisons of the correlated ROC curves performed using DeLong’s test. Bold values indicate statistical significance (*p* < 0.05). Abbreviations: AUC, area under the curve; CI, confidence interval; CPR, cerebroplacental ratio; CPRR, cerebroplacental–renal ratio; CPUR, cerebroplacental–uterine ratio; ΔAUC, difference in area under the curve; SE, standard error.

**Table 5:** Univariable and multivariable logistic regression analyses for adverse perinatal outcome, with odds ratios for CPR, CPUR, and CPRR expressed per 0.1-unit decrease.

Variable/Model	Crude OR (95% CI)	*p*	Adjusted OR (95% CI)	*p*	Model Statistics
**Univariable analysis**					
GA at US	0.842 (0.600–1.183)	0.322			
Preeclampsia	7.639 (2.656–21.970)	**<0.001**			
UA PI	1.419 (1.009–1.995)	**0.044**			
Mean UtA PI	1.224 (1.030–1.454)	**0.022**			
Right RA PI	1.335 (1.106–1.610)	**0.003**			
CPR	1.149 (0.959–1.378)	0.133			
CPUR	1.237 (1.070–1.430)	**0.004**			
CPRR	1.510 (1.239–1.840)	**<0.001**			
**Multivariable analysis**					
Model 1: CPR-based model					Nagelkerke R^2^ = 0.226; AIC = 125.410
GA at US			0.957 (0.653–1.402)	0.820	
Preeclampsia			9.310 (2.687–32.249)	**<0.001**	
CPR			0.919 (0.731–1.155)	0.471	
Model 2: CPUR-based model					Nagelkerke R^2^ = 0.233; AIC = 124.815
GA at US			0.968 (0.660–1.421)	0.869	
Preeclampsia			5.716 (1.763–18.529)	**0.004**	
CPUR			1.090 (0.927–1.281)	0.296	
Model 3: CPRR-based model					Nagelkerke R^2^ = 0.377; AIC = 110.647
GA at US			0.911 (0.602–1.379)	0.661	
Preeclampsia			4.518 (1.443–14.147)	**0.010**	
CPRR			1.423 (1.162–1.743)	**0.001**	

Bold values indicate statistical significance (*p* < 0.05). All multivariable models were adjusted for gestational age at ultrasound assessment and diagnosis group. Odds ratios for GA at US refer to each 1-week increase. Odds ratios for UA PI, mean UtA PI, and right RA PI refer to each 0.1-unit increase, whereas odds ratios for CPR, CPUR, and CPRR refer to each 0.1-unit decrease. Gestational hypertension was used as the reference category for preeclampsia. AIC was calculated as −2 log likelihood + 2k, where k represents the number of estimated model parameters, including the intercept. Abbreviations: AIC, Akaike information criterion; CI, confidence interval; CPR, cerebroplacental ratio; CPRR, cerebroplacental–renal ratio; CPUR, cerebroplacental–uterine ratio; GA, gestational age; OR, odds ratio; PI, pulsatility index; RA, renal artery; UA, umbilical artery; US, ultrasound; UtA, uterine artery.

## Data Availability

The data presented in this study are available on request from the corresponding author due to privacy and ethical restrictions.

## References

[1] Magee L.A., Brown M.A., Hall D.R., Gupte S., Hennessy A., Karumanchi S.A., Kenny L.C., McCarthy F., Myers J., Poon L.C. (2022). The 2021 International Society for the Study of Hypertension in Pregnancy classification, diagnosis & management recommendations for international practice. Pregnancy Hypertens..

[2] Berhe A.K., Ilesanmi A.O., Aimakhu C.O., Mulugeta A. (2019). Effect of pregnancy induced hypertension on adverse perinatal outcomes in Tigray regional state, Ethiopia: A prospective cohort study. BMC Pregnancy Childbirth.

[3] Bajpai D., Popa C., Verma P., Dumanski S., Shah S. (2023). Evaluation and Management of Hypertensive Disorders of Pregnancy. Kidney360.

[4] Zarean E., Azami N., Shahshahan Z. (2022). Predictive Value of Middle Cerebral Artery to Umbilical Artery Pulsatility Index Ratio for Neonatal Outcomes in Hypertensive Disorders of Pregnancy. Adv. Biomed. Res..

[5] Phipps E.A., Thadhani R., Benzing T., Karumanchi S.A. (2019). Pre-eclampsia: Pathogenesis, novel diagnostics and therapies. Nat. Rev. Nephrol..

[6] Bakrania B.A., Spradley F.T., Drummond H.A., LaMarca B., Ryan M.J., Granger J.P. (2020). Preeclampsia: Linking Placental Ischemia with Maternal Endothelial and Vascular Dysfunction. Compr. Physiol..

[7] Khalil A., Morales-Roselló J., Townsend R., Morlando M., Papageorghiou A., Bhide A., Thilaganathan B. (2016). Value of third-trimester cerebroplacental ratio and uterine artery Doppler indices as predictors of stillbirth and perinatal loss. Ultrasound Obstet. Gynecol..

[8] Konwar R., Basumatari B., Dutta M., Mahanta P., Saikia A., Uk R. (2021). Role of Doppler Waveforms in Pregnancy-Induced Hypertension and Its Correlation with Perinatal Outcome. Cureus.

[9] Lin X., Liu L.L., Zheng L.J., Yang C.Y. (2023). Evaluation of Doppler indices (MCA & UA) and fetal outcomes: A retrospective case-control study in women with hypertensive disorders of pregnancy. J. Matern. Fetal Neonatal Med..

[10] MacDonald T.M., Hui L., Robinson A.J., Dane K.M., Middleton A.L., Tong S., Walker S.P. (2019). Cerebral-placental-uterine ratio as novel predictor of late fetal growth restriction: Prospective cohort study. Ultrasound Obstet. Gynecol..

[11] Agaoglu Z., Tanacan A., Ipek G., Peker A., Ozturk Agaoglu M., Ozkavak O.O., Kara O., Sahin D. (2024). The role of the cerebro-placental-uterine ratio in predicting composite adverse perinatal outcomes in patients with pregnancy-induced hypertension. Pregnancy Hypertens..

[12] Andronikidi P.E., Orovou E., Mavrigiannaki E., Athanasiadou V., Tzitiridou-Chatzopoulou M., Iatrakis G., Grapsa E. (2024). Placental and Renal Pathways Underlying Pre-Eclampsia. Int. J. Mol. Sci..

[13] Bahser N., Godehardt E., Hess A.P., Blume C. (2014). Examination of intrarenal resistance indices indicate the involvement of renal pathology as a significant diagnostic classifier of preeclampsia. Am. J. Hypertens..

[14] Ogunmoroti O.A., Ayoola O.O., Makinde O.N., Idowu B.M. (2015). Maternal renal artery Doppler sonographic changes in pregnancy-induced hypertension in South West Nigeria. Niger. Med. J..

[15] Isiaka Olanrewaju Akintunde T., Suwaid M.A., Adamu M.Y., Musa A., Nafiah Abolanle T., Ismail A. (2024). Comparative sonographic assessment of renal volume and arterial Doppler velocimetric indices among women with pregnancy-induced hypertension and normotensive controls in Northern Nigeria. Ultrasound.

[16] Bellos I., Pergialiotis V. (2020). Doppler parameters of renal hemodynamics in women with preeclampsia: A systematic review and meta-analysis. J. Clin. Hypertens..

[17] von Elm E., Altman D.G., Egger M., Pocock S.J., Gøtzsche P.C., Vandenbroucke J.P. (2007). Strengthening the Reporting of Observational Studies in Epidemiology (STROBE) statement: Guidelines for reporting observational studies. BMJ.

[18] The American College of Obstetricians and Gynecologists (2020). Gestational Hypertension and Preeclampsia: ACOG Practice Bulletin, Number 222. Obstet. Gynecol..

[19] Villar J., Cheikh Ismail L., Victora C.G., Ohuma E.O., Bertino E., Altman D.G., Lambert A., Papageorghiou A.T., Carvalho M., Jaffer Y.A. (2014). International standards for newborn weight, length, and head circumference by gestational age and sex: The Newborn Cross-Sectional Study of the INTERGROWTH-21st Project. Lancet.

[20] Bhide A., Acharya G., Baschat A., Bilardo C.M., Brezinka C., Cafici D., Ebbing C., Hernandez-Andrade E., Kalache K., Kingdom J. (2021). ISUOG Practice Guidelines (updated): Use of Doppler velocimetry in obstetrics. Ultrasound Obstet. Gynecol..

[21] Ciobanu A., Wright A., Syngelaki A., Wright D., Akolekar R., Nicolaides K.H. (2019). Fetal Medicine Foundation reference ranges for umbilical artery and middle cerebral artery pulsatility index and cerebroplacental ratio. Ultrasound Obstet. Gynecol..

[22] Faul F., Erdfelder E., Buchner A., Lang A.G. (2009). Statistical power analyses using G*Power 3.1: Tests for correlation and regression analyses. Behav. Res. Methods.

[23] Karabay G., Bayraktar B., Seyhanli Z., Filiz A.A., Tokgoz Cakir B., Aktemur G., Tonyali N.V., Agaoglu R.T., Kocaoglu G., Karabay U. (2025). Evaluation of Conventional and Combined Doppler Parameters in Preeclampsia: Diagnostic and Prognostic Insights. J. Clin. Med..

[24] Lodge J., Flatley C., Kumar S. (2021). The fetal cerebroplacental ratio in pregnancies complicated by hypertensive disorders of pregnancy. Aust. N. Z. J. Obstet. Gynaecol..

[25] Oğuz Y., Ağaoğlu R.T., Ulusoy C.O., Öztürk Ö., Özgürlük İ., Soysal Ç., Yılmaz Vural Z., Yakut Yücel K. (2024). A new Doppler index, cerebro-placental-uterine ratio, and fetal cardiac parameters in early onset preeclampsia. J. Clin. Ultrasound.

[26] Morales-Roselló J., Buongiorno S., Loscalzo G., Abad García C., Cañada Martínez A.J., Perales Marín A. (2020). Does Uterine Doppler Add Information to the Cerebroplacental Ratio for the Prediction of Adverse Perinatal Outcome at the End of Pregnancy?. Fetal Diagn. Ther..

[27] Graupner O., Meister M., Lecker L., Karim-Payab S., Franz C., Carow J., Enzensberger C. (2023). Role of the cerebro-placental-uterine ratio in predicting adverse perinatal outcome in low-risk pregnancies at term. Arch. Gynecol. Obstet..

[28] Salehi M.G., Shobeiri E., Naleini F., Bazargan M.S. (2021). Diagnostic value of doppler ultrasound indices of maternal renal interlobar vasculature in the prediction of preeclampsia. J. Med. Life.

